# Risk factors for and prognostic values of postoperative acute kidney injury after pancreaticoduodenectomy for pancreatic ductal adenocarcinoma: A retrospective, propensity score‐matched cohort study of 1312 patients

**DOI:** 10.1002/cam4.5543

**Published:** 2022-12-15

**Authors:** Yuchen Ji, Yiran Zhou, Ziyun Shen, Haoda Chen, Shulin Zhao, Xiaxing Deng, Baiyong Shen

**Affiliations:** ^1^ Department of General Surgery, Pancreatic Disease Center, Ruijin Hospital Shanghai Jiao Tong University School of Medicine Shanghai China; ^2^ Institute of Translational Medicine Shanghai Jiao Tong University Shanghai China; ^3^ Research Institute of Pancreatic Disease Shanghai Jiao Tong University School of Medicine Shanghai China; ^4^ State Key Laboratory of Oncogenes and Related Genes Shanghai China

**Keywords:** 30‐day mortality, long‐term survival, pancreaticoduodenectomy, postoperative acute kidney injury

## Abstract

**Background:**

While an association between postoperative acute kidney injury (AKI) and adverse events exists, the incidence and impact of postoperative AKI after pancreaticoduodenectomy for pancreatic ductal adenocarcinoma remain unclear. This study aimed to diagnose AKI and investigate the risk factors for and prognostic value of postoperative AKI.

**Methods:**

Clinical characteristics of patients who underwent pancreaticoduodenectomy between 2013 and 2020 at a high‐volume centre were collected retrospectively. The Kidney Disease Improving Global Outcomes criteria were used to diagnose AKI. A 1:2 propensity score matching (PSM) was used to minimise bias between the AKI and non‐AKI groups. Short‐term surgical and long‐term survival outcomes were compared between groups. Multivariate logistic regression analysis assessed the independent risk factors for AKI development, major complications, and 30‐day mortality.

**Results:**

Postoperative AKI occurred in 10.7% of 1312 patients. Total bilirubin level > 250 μmol/L (odds ratio [OR]: 3.24; *p* < 0.001), estimated glomerular filtration rate < 60 ml/min/1.73 m^2^ (OR: 2.30; *p* = 0.048), and intraoperative estimated blood loss >1000 ml (OR: 2.96; *p* = 0.001) were independent risk factors for postoperative AKI. After PSM, higher incidences of major complications (*p* < 0.001) and 30‐day mortality (*p* < 0.001) were observed in the AKI group than in the non‐AKI group. There was no difference in long‐term overall survival outcomes between both groups (*p* = 0.535). AKI was an independent predictor of major complications (OR: 3.06; *p* < 0.001) and 30‐day mortality (OR: 2.87; *p* = 0.034).

**Conclusions:**

Postoperative AKI is common after pancreaticoduodenectomy for pancreatic ductal adenocarcinoma and has a predictive effect on major complications and 30‐day mortality. Therefore, prevention and proper management of postoperative AKI are required in clinical practice.

## Graphical abstract

Acute kidney injury is an independent predictor of major complications and 30‐day mortality after pancreaticoduodenectomy for pancreatic ductal adenocarcinoma.

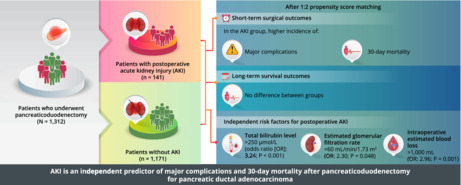

## 1 INTRODUCTION

Currently, pancreaticoduodenectomy (PD) remains a standard treatment for pancreatic ductal adenocarcinomas (PDAC) in the head of the pancreas.^1^ Although the associated postoperative mortality decreases to <5% when performed in most high‐volume institutions,^2^, ^3^ PD has a high morbidity rate and remains a dangerous abdominal surgery. Various mortality‐related and highly associated risk factors and postoperative complications^4^, ^5^, ^6^, ^7^, ^8^ have been studied to improve perioperative management. To date, acute kidney injury (AKI) remains minimally studied. Further exploration is required for clinical guidance.

Studies have revealed that AKI was associated with an increased mortality rate after cardiac surgery.^9^, ^10^, ^11^ However, the incidence and impact of AKI after non‐cardiac surgeries have been infrequently explored. A previous systematic review observed that AKI incidence after major abdominal surgeries was 13.4% (10.9–16.4%).^12^ In addition, liver surgeries, such as resection for carcinoma^13^, ^14^ and transplantation,^15^, ^16^ had the highest proportion among all abdominal surgeries in revealing the outcomes and risk factors of AKI. Yet, whether AKI after pancreatic surgery worsens short‐term outcomes has been rarely studied, and risk factors have not been well‐established. Furthermore, the impact of postoperative AKI on long‐term survival outcomes remains unknown for patients with PDAC who underwent PD.

The definition of AKI was developed over several decades. Using different diagnostic standards, the prevalence of postoperative AKI varied greatly. In 2012, the Kidney Disease: Improving Global Outcomes (KDIGO) Foundation proposed a widely used guideline that accepted the existing criteria for defining AKI and implemented simplified criteria for practice.^17^

In routine clinical managements after PD, surgeons pay the most attention to high‐risk complications, such as fistula, haemorrhage and infections. Postoperative AKI sometimes can be covert and easily ignored, with only slight symptoms or few level changes in biochemical indicators. There were also few studies highlighting the significance of AKI after PD surgeries. We assumed that the occurrence could increase the risk of postoperative morbidity and mortality, and this study aimed to study its risk factors and impacts on both short‐term surgical and long‐term survival outcomes.

## 2 METHODS

### 2.1 Patient criteria and data collection

Data from patients who underwent the classic Whipple procedure with standard lymphadenectomy for PDAC between 1 January 2013 and 31 December 2020 at our institute were retrospectively collected, and the tumor pathology was confirmed. We excluded patients younger than 18 years and those with pre‐existing pancreatic surgery, evidence of metastasis, locally advanced or borderline unresectable tumors, and additional organ resection (such as the kidney or colon).

Preoperative clinical data included patient demographics (age, sex, body mass index, American Society of Anesthesiologists [ASA] class, comorbidity, and habits), preoperative laboratory data (white blood cell count, haemoglobin level, total bilirubin [Tbil] level, albumin level, serum creatinine [SCr] level, estimated glomerular filtration rate [eGFR]), and preoperative biliary drainage status. Intraoperative data included the surgical type (open PD or robot‐assisted PD), venous resection (portal or superior mesenteric vein), operative time, estimated blood loss, intraoperative transfusion, and stage (T or N stage). Postoperative surgical complications (30‐day mortality, reintervention, clinically relevant postoperative pancreatic fistula [CR‐POPF], biliary leak [BL], intestinal leak [IL], post‐pancreatectomy haemorrhage [PPH], delayed gastric emptying [DGE], and intra‐abdominal infection) and postoperative hospital stay were also recorded to identify short‐term outcomes.

### 2.2 Definitions of short‐term surgical outcomes

Short‐term mortality was defined as death within 30 days postoperatively. CR‐POPF,^18^ PPH,^19^ and DGE^20^ were diagnosed according to the International Study Group of Pancreatic Surgery. BL and IL, which commonly require intervention such as peritoneal lavage or computed tomography‐guided abdominal drainage, were defined as the observation of abnormal drainage confirmed through radiographic, endoscopic, or other interventional techniques. Intra‐abdominal infection was defined as persistent symptoms of postoperative fever with positive culture results or radiologic evidence. Postoperative hospital stay was the number of days after surgery until discharge. All complications were further assessed according to the Clavien–Dindo classification (CDC)^21^ and the most severe one had the highest CDC grade among all complications that occurred to the same patient. Major complications were defined for the most severe ones if their CDC grades were classified as III, IV, or V.

### 2.3 Follow‐up

After PD for PDAC, patients were followed up at regular intervals via telephone every 3 months, and when the patient died, or contact was lost, the follow‐up ended. We focused on overall survival (OS), the time from PD to death attributable to any cause. Survival data were censored at the date of the last follow‐up, and our latest follow‐up occurred in March 2022.

### 2.4 Diagnostic criteria and severity staging for postoperative acute kidney injury

The KDIGO system was used during this study. A blood test was conducted on the admission day to define baseline SCr values,^22^ and the Modification of Diet in Renal Disease Equation was used to calculate the eGFR.^23^ Patients were considered to have potential chronic kidney disease when the eGFR was <60 ml/min/1.73 m^2^, regardless of the medical history.^24^ Postoperative AKI was diagnosed by increased SCr of ≥0.3 mg/dL (26.5 μmol/L) within 48 hr or an increase to at least 1.5 times the baseline value within 7 days postoperatively.^25^ The urine volume diagnostic criteria were excluded due to incomplete data. Based on the ratio between the highest SCr level after PD and the baseline, severity stages were as follows: stage 1, 1.5–1.9 times baseline or at least a 0.3 mg/dL increase; stage 2, 2–2.9 times baseline; and stage 3, at least 3 times an increase in the baseline SCr level to 353.6 mmol/L (or 4 mg/dl), or renal replacement therapy initiation.

### 2.5 Statistical analysis

Statistical analysis was performed using SPSS version 26.0 (IBM Corp.). All the tests with a two‐tailed P < 0.05 were considered significant. Categorical variables were presented as numbers (percentages) and compared using the Pearson chi‐square or Fisher exact test. Continuous variables were presented as means ± standard deviations using Student's *t*‐test when they are normally distributed or medians (interquartile ranges) using the Mann–Whitney *U* test when they are not. Univariate independent risk factors of postoperative AKI, major complications, and 30‐day mortality were first analysed, and those with *p* < 0.05 were entered into the multivariate logistic regression model, and we recorded the odds ratio (OR) and 95% confidence interval (CIs). Furthermore, preoperative and intraoperative risk factors were analysed for AKI development; AKI status was further included in the analysis for major complications, and 30‐day mortality also included postoperative complications. The entire AKI and non‐AKI cohorts were unbalanced. Patients who developed AKI might have been older and had worse eGFRs, and other different baseline characteristics, leading to increased morbidity and mortality. To reduce selection bias and make the AKI status the only exposure, we used R software (R Core Team), SPSS Statistics Essentials for R, and SPSS PS Matching to perform 1:2 nearest‐neighbour propensity score matching (PSM); calliper widths with a standard deviation of 0.05 were allowed. PSM was created using the following baseline characteristics: age, sex, body mass index, ASA class, diabetes mellitus, hypertension, cardiovascular disease, haemoglobin level, TBil level, albumin, baseline SCr level, eGFR, surgical type, venous resection, operative time, intraoperative estimated blood loss, and transfusion. After PSM, all preoperative and intraoperative factors were well‐balanced between the control and observational groups, and a direct causal relationship of AKI and morbidities was analysed. Subsequently, survival curves were constructed using the Kaplan–Meier (KM) method with the log‐rank test, and OS was analysed after PSM. Patients who died within 30 days postoperatively or were lost during regular follow‐up were excluded from the KM analysis.

## 3 RESULTS

### 3.1 Study population

During the 8‐year study period, 1312 patients were included according to our selection criteria; 141 (10.7%) developed postoperative AKI. Among the entire AKI group, the AKI severity stage was stage 1 for 112 cases (79.4%), stage 2 for 21 (14.9%), and stage 3 for 8 (5.7%). According to the treatment data, no patient with stage 3 was admitted to the intensive care unit or underwent renal replacement therapy. All AKI patients underwent only clinical observation or fluid control therapy. Overall, 111 (78.7%) of the AKI cases occurred during the first 2 days after PD, and the number continued to decrease to 6 (4.3%) during the final 2 days within a week. Thirty‐day mortality and major complications were significantly correlated with the AKI severity stages 1, 2, and 3 (5 [4.5%], 3 [14.3%], and 3 [37.5%], respectively, for 30‐day mortality, *p* = 0.002; 11 [9.8%], 7 [33.3%], and 3 [37.5%], respectively, for major complications, *p* = 0.004). The results revealed no significant association between 30‐day mortality and major complications and the time of postoperative AKI occurrence.

### 3.2 Preoperative and intraoperative characteristics of the cohort before and after propensity score matching

In the entire cohort, patients with postoperative AKI were older (65.74 ± 8.78 years vs. 63.10 ± 9.46 years; *p* = 0.002), had a higher ASA class (*p* = 0.003), and were more likely to have a history of cardiovascular disease than those without postoperative AKI (*p* = 0.008). They also had lower haemoglobin and serum albumin levels and higher white blood cell counts and eGFR than those without postoperative AKI. Furthermore, the preoperative biliary drainage status significantly differed (58 [41.1%] vs. 352 [30.1%]; *p* = 0.01), which was consistent with the higher TBil level of those with postoperative AKI compared to those without (*p* < 0.001). There were no significant differences in patient characteristics such as sex proportion, body mass index, comorbidities including diabetes mellitus and hypertension, smoking and alcohol habits, baseline SCr level, surgical type, venous resection, operative time, estimated blood loss, intraoperative transfusion, and tumor stage between both groups. After PSM, a well‐balanced cohort was created, with the non‐AKI group (228 patients) as the control group and the AKI group as the observational group (114 patients). Preoperative and intraoperative characteristics are presented in Table 1. Baseline data were not significantly different between groups after PSM.

**TABLE 1:** Pre‐ and intraoperative baseline characteristics before and after PSM

Variables	Before PSM			After PSM		
	Without AKI (*n* = 1171)	With AKI (*n* = 141)	*p*‐value	Without AKI (*n* = 228)	With AKI (*n* = 114)	*p*‐value
Age (mean (SD)), years	63.10 (9.46)	65.74 (8.78)	0.002	64.81 (10.17)	65.58 (8.68)	0.491
Male gender, *n* (%)	729 (62.3)	100 (70.9)	0.054	150 (65.8)	78 (68.4)	0.715
BMI (mean (SD)), kg/m^2^	22.75 (2.98)	22.71 (3.46)	0.876	22.50 (3.00)	22.70 (3.38)	0.581
ASA class, *n* (%)						
I	526 (44.9)	49 (34.8)	0.003	87 (38.2)	41 (36.0)	0.924
II	507 (43.3)	62 (44.0)		100 (43.9)	52 (45.6)	
III + IV	138 (11.8)	30 (21.3)		41 (18.0)	21 (18.4)	
Comorbidity						
Diabetes mellitus, *n* (%)	271 (23.1)	36 (25.5)	0.598	53 (23.2)	30 (26.3)	0.624
Hypertension, *n* (%)	430 (36.7)	64 (45.4)	0.055	89 (39.0)	48 (42.1)	0.668
Cardiovascular disease, *n* (%)	92 (7.9)	21 (14.9)	0.008	24 (10.5)	14 (12.3)	0.761
Habit						
Smoke, *n* (%)	317 (27.1)	42 (29.8)	0.560	65 (28.5)	32 (28.1)	1.000
Alcohol, *n* (%)	227 (19.4)	36 (25.5)	0.107	45 (19.7)	30 (26.3)	0.212
Preoperative laboratory data						
WBC (median, IQR), ×10^9^/L	5.80 (4.70–7.00)	6.10 (5.08–7.84)	0.032	6.07 (4.90–7.20)	6.10 (4.98–7.70)	0.664
Hb (median, IQR), g/L	127 (116–137)	125 (112–134)	0.050	126 (113–138)	126 (112–135)	0.495
TBil (median, IQR), μmol/L	38.2 (13.7–126.6)	118.2 (17.8–202.9)	<0.001	69.2 (14.5–166.2)	67.5 (15.9–155.3)	0.685
ALB (median, IQR), g/L	38 (35–41)	37 (34–41)	0.003	38 (34–41)	37 (34–41)	0.855
Baseline sCr (median, IQR), μmol/L	68 (58–78)	67 (54–78)	0.159	67 (55–76)	70 (57–78)	0.219
eGFR (median, IQR), ml/min/1.73 m^2^	98.93 (84.57–114.75)	102.39 (84.74–132.47)	0.025	102.84 (88.54–120.23)	98.46 (83.84–122.43)	0.333
Preoperative biliary drainage, *n* (%)	352 (30.1)	58 (41.1)	0.010	83 (36.4)	43 (37.7)	0.905
Surgical type, RPD, *n* (%)	167 (14.3)	18 (12.8)	0.723	22 (9.6)	14 (12.3)	0.575
Venous resection, *n* (%)	197 (16.8)	29 (20.6)	0.320	46 (20.2)	24 (21.1)	0.962
Intraoperative data						
Operative time (median, IQR), min	300 (255–360)	310 (260–360)	0.221	305 (270–360)	310 (255–360)	0.969
Estimated blood loss (median, IQR), ml	300 (200–500)	310 (260–360)	0.070	400 (200–600)	350 (200–600)	0.461
Intraoperative transfusion (median, IQR), ml	800 (0–1200)	800 (0–1450)	0.463	800 (0–1200)	800 (0–1200)	0.962
Stage						
T stage, *n* (%)						
T1	2 (0.2)	1 (0.7)	0.604	0 (0.0)	1 (0.9)	0.531
T2	98 (8.4)	11 (7.8)		21 (9.2)	10 (8.8)	
T3	888 (75.8)	109 (77.3)		175 (76.8)	89 (78.1)	
T4	183 (15.6)	20 (14.2)		32 (14.0)	14 (12.3)	
N stage, *n* (%)						
N0	563 (48.1)	68 (48.2)	0.801	97 (42.5)	60 (52.6)	0.169
N1	447 (38.2)	51 (36.2)		94 (41.2)	36 (31.6)	
N2	161 (13.7)	22 (15.6)		37 (16.2)	18 (15.8)	

Abbreviations: ALB, albumin; ASA, American Society of Anesthesiologists; BMI, body mass index; eGFR, estimated glomerular filtration rate; Hb, hemoglobin; IQR, interquartile range; RPD, robotic pancreaticoduodenectomy; sCr, serum creatinine; SD, standard deviation; TBil, total bilirubin; WBC, white blood cell.

### 3.3 Postoperative complications and 30‐day mortality after propensity score matching

Comparisons of postoperative short‐term outcomes are presented in Table 2. After PSM, 30‐day mortality (11 [9.6%] vs. 2 [0.9%]; *p* < 0.001) and major complications (20 [17.5%] vs. 12 [5.3%]; *p* = 0.001) were significantly different between groups. Patients also experienced an increased incidence of CR‐POPF (38 [33.3%] vs. 39 [17.1%]; *p* = 0.001), especially grade C, BL (23 [20.2%] vs. 17 [7.5%]; *p* = 0.001), IL (5 [4.4%] vs. 1 [0.4%]; *p* = 0.029), PPH grades B and C (13 [11.4%] vs. 5 [2.2%]; *p* = 0.001), and intra‐abdominal infections (40 [35.1%] vs. 47 [20.6%]; *p* = 0.006) when postoperative AKI occurred. The reintervention rates during the hospital stay (13 [11.4%] vs. 7 [3.1%]; *p* = 0.004) also differed between groups.

**TABLE 2:** The impact of postoperative AKI on surgical complications and 30‐day mortality before and after PSM

Variables	Before PSM			After PSM		
	Without AKI (*n* = 1171)	With AKI (*n* = 141)	*p*‐value	Without AKI (*n* = 228)	With AKI (*n* = 114)	*p*‐value
CR‐POPF, *n* (%)	195 (16.7)	45 (31.9)	<0.001	39 (17.1)	38 (33.3)	0.001
Grade B, *n* (%)	180 (15.4)	30 (21.3)	0.092	36 (15.8)	23 (20.2)	0.390
Grade C, *n* (%)	15 (1.3)	15 (10.6)	<0.001	3 (1.3)	15 (13.2)	<0.001
Biliary leak, *n* (%)	87 (7.4)	28 (19.9)	<0.001	17 (7.5)	23 (20.2)	0.001
Intestinal leak, *n* (%)	10 (0.9)	5 (3.5)	0.015	1 (0.4)	5 (4.4)	0.029
DGE grade B & C, *n* (%)	34 (2.9)	4 (2.8)	1.000	6 (2.6)	3 (2.6)	1.000
PPH, *n* (%)	39 (3.3)	20 (14.2)	<0.001	9 (3.9)	17 (14.9)	0.001
Grade A, *n* (%)	11 (0.9)	7 (5.0)	<0.001	4 (1.8)	4 (3.5)	0.527
Grade B & C, *n* (%)	28 (2.4)	13 (9.2)	<0.001	5 (2.2)	13 (11.4)	0.001
DSA, *n* (%)	11 (0.9)	2 (1.4)	0.926	3 (1.3)	2 (1.8)	1.000
Relaparotomy, *n* (%)	13 (1.1)	8 (5.7)	<0.001	1 (0.4)	8 (7.0)	0.001
DSA+ Relaparotomy, *n* (%)	4 (0.3)	3 (2.1)	0.032	1 (0.4)	3 (2.6)	0.213
Intra‐abdominal infections, *n* (%)	224 (19.1)	48 (34.0)	<0.001	47 (20.6)	40 (35.1)	0.006
CDC for major complications, *n* (%)						
I	749 (64.0)	60 (42.6)	<0.001	144 (63.2)	47 (41.2)	<0.001
II	361 (30.8)	60 (42.6)		72 (31.6)	47 (41.2)	
III	21 (1.8)	2 (1.4)		6 (2.6)	1 (0.9)	
IV	24 (2.0)	8 (5.7)		4 (1.8)	8 (7.0)	
V	16 (1.4)	11 (7.8)		2 (0.9)	11 (9.6)	
CDC≥III, *n*(%)	61 (5.2)	21 (14.9)	<0.001	12 (5.3)	20 (17.5)	0.001
Reintervention, *n* (%)	37 (3.2)	13 (9.2)	0.001	7 (3.1)	13 (11.4)	0.004
30‐day mortality, *n* (%)	16 (1.4)	11 (7.8)	<0.001	2 (0.9)	11 (9.6)	<0.001
Postoperative hospital stay (median, IQR), days	18 (14–25)	21 (15–28)	0.012	18 (14–25)	21 (15–28)	0.188

Abbreviations: CDC, Clavien‐Dindo classification; CR‐POPF, clinically relevant postoperative pancreatic fistula; DGE, delayed gastric emptying; DSA, digital subtraction angiography; IQR, interquartile range; PPH, post‐pancreatectomy hemorrhage.

### 3.4 Potential risk factors for postoperative acute kidney injury

We conducted univariate and multivariate analyses to determine potential risk factors for postoperative AKI development in patients who underwent PD (Table 3). During the univariate analysis, age 70 years or older, male sex, ASA class III, hypertension, cardiovascular disease, TBil level > 250 μmol/L, albumin level < 35 mg/L, eGFR <60 ml/min/1.73 m^2^, preoperative biliary drainage, operative time > 300 min, and intraoperative estimated blood loss >1000 ml were significantly associated with AKI development. During the multivariate logistic regression analysis, however, only a TBil level > 250 μmol/L (OR: 3.24; 95% CI: 1.83–5.74; *p* < 0.001), eGFR <60 ml/min/1.73 m^2^ (OR: 2.30; 95% CI: 1.01–5.26; *p* = 0.048), and estimated blood loss >1000 ml (OR: 2.96; 95% CI: 1.53–5.28; *p* = 0.001) were independent risk factors for postoperative AKI.

**TABLE 3:** Risk factors of postoperative AKI: univariate and multivariate analyses

Risk factors	Univariate analysis		Multivariate analysis	
	OR (95% CI)	*p*‐value	OR (95% CI)	*p*‐value
Age ≥ 70 years	1.62 (1.12–2.34)	0.011	1.31 (0.88–1.94)	0.186
Male gender	1.48 (1.01–2.17)	0.045	1.34 (0.90–2.00)	0.144
BMI > 28 kg/m^2^	1.94 (0.98–3.82)	0.056		
ASA class≥III	2.02 (1.30–3.14)	0.002	1.53 (0.70–3.36)	0.290
Diabetes mellitus	1.14 (0.76–1.70)	0.527		
Hypertension	1.43 (1.01–2.04)	0.046	1.15 (0.78–1.68)	0.487
Cardiovascular disease	2.05 (1.23–3.42)	0.006	1.21 (0.49–2.98)	0.681
Smoke	1.14 (0.78–1.68)	0.495		
Alcohol	1.43 (0.95–2.14)	0.086		
WBC > 10 × 10^9^/L	1.49 (0.74–2.99)	0.260		
Hb < 90 g/L	1.23 (0.47–3.20)	0.672		
TBil > 250 μmol/L	3.90 (2.29–6.64)	<0.001	3.24 (1.83–5.74)	<0.001
ALB < 35 g/L	1.68 (1.15–2.46)	0.008	1.23 (0.81–1.87)	0.342
Baseline sCr > 133 μmol/L	2.09 (0.44–9.95)	0.354		
eGFR < 60 ml/min/1.73 m^2^	2.89 (1.33–6.27)	0.007	2.30 (1.01–5.26)	0.048
Preoperative biliary drainage	1.63 (1.14–2.33)	0.008	1.31 (0.89–1.93)	0.170
Surgical type, RPD	0.88 (0.52–1.48)	0.630		
Venous resection	1.28 (0.83–1.98)	0.267		
Operative time > 300 min	1.52 (1.07–2.17)	0.019	1.40 (0.96–2.02)	0.078
Estimated blood loss > 1000 ml	3.72 (2.02–6.84)	<0.001	2.96 (1.53–5.28)	0.001
Intraoperative transfusion > 1000 ml	1.37 (0.94–1.99)	0.101		

Abbreviations: ALB, albumin; ASA, American Society of Anesthesiologists; BMI, body mass index; eGFR, estimated glomerular filtration rate; Hb, hemoglobin; RPD, robotic pancreaticoduodenectomy; sCr, serum creatinine; TBil, total bilirubin; WBC, white blood cell.

### 3.5 Risk factors of major complications and 30‐day mortality after pancreaticoduodenectomy

Risk factors are displayed in Table S1 for major complications and Table 4 for 30‐day mortality. In the univariate analysis, AKI (OR: 3.18; 95% CI: 1.87–5.41; *p* < 0.001), diabetes mellitus, comorbidities, and white blood cell count >10 × 10^9^/L were associated with major complications. After the multivariate regression, only AKI (OR: 3.06; 95% CI: 1.76–5.32; *p* < 0.001) and diabetes mellitus (OR: 3.57; 95% CI: 2.25–5.67; *p* < 0.001) remained independent predictors for major complications.

**TABLE 4:** Univariate and multivariate analyses of risk factors for 30‐day mortality

Risk factors	Univariate analysis		Multivariate analysis	
	OR (95% CI)	*p*‐value	OR (95% CI)	*p*‐value
Age ≥ 70 years	3.08 (1.43–6.62)	0.004	4.08 (1.56–10.64)	0.004
Male gender	1.17 (0.52–2.62)	0.705		
BMI > 28 kg/m^2^	2.69 (0.79–9.21)	0.114		
ASA class≥III	1.19 (0.41–3.48)	0.752		
Diabetes mellitus	0.93 (0.37–2.34)	0.884		
Hypertension	1.14 (0.53–2.48)	0.738		
Cardiovascular disease	1.34 (0.40–4.51)	0.641		
Smoke	1.58 (0.72–3.48)	0.258		
Alcohol	1.70 (0.74–3.93)	0.214		
WBC > 10 × 10^9^/L	3.37 (1.13–10.05)	0.029	2.27 (0.57–8.96)	0.244
Hb < 90 g/L	4.34 (1.25–15.06)	0.021	2.04 (0.39–10.54)	0.395
TBil > 250 μmol/L	2.11 (0.62–7.16)	0.233		
ALB < 35 g/L	1.76 (0.78–3.97)	0.171		
Baseline sCr > 133 μmol/L	5.45 (0.67–44.63)	0.114		
eGFR < 60 ml/min/1.73 m^2^	2.94 (0.67–12.93)	0.153		
Preoperative biliary drainage	2.08 (0.97–4.46)	0.061		
Surgical type, RPD	1.06 (0.36–3.10)	0.914		
Venous resection	1.38 (0.55–3.47)	0.489		
Operative time > 300 min	2.26 (1.01–5.06)	0.048	2.17 (0.81–5.80)	0.122
Estimated blood loss > 1000 ml	0.88 (0.12–6.58)	0.898		
Intraoperative transfusion > 1000 ml	1.90 (0.88–4.14)	0.105		
Postoperative AKI	6.11 (2.78–13.44)	<0.001	2.87 (1.09–7.58)	0.034
CR‐POPF	4.33 (2.01–9.33)	<0.001	0.96 (0.34–2.70)	0.933
Biliary leak	5.56 (2.44–12.68)	<0.001	1.10 (0.36–3.36)	0.864
Intestinal leak	40.51 (13.23–124.07)	<0.001	11.60 (2.66–50.71)	0.001
PPH grade B and C	28.76 (12.31–67.21)	<0.001	11.25 (3.60–35.13)	<0.001
Intra‐abdominal infections	11.71 (4.90–28.00)	<0.001	3.46 (1.22–9.87)	0.020
DGE grade B and C	1.30 (0.17–9.82)	0.801		

Abbreviations: AKI, acute kidney injury; ALB, albumin; ASA, American Society of Anesthesiologists; BMI, body mass index; CR‐POPF, clinically relevant postoperative pancreatic fistula; DGE, delayed gastric emptying; eGFR, estimated glomerular filtration rate; Hb, hemoglobin; PPH, post‐pancreatectomy hemorrhage; RPD, robotic pancreaticoduodenectomy; sCr, serum creatinine; TBil, total bilirubin; WBC, white blood cell.

During the univariate analysis, 30‐day mortality after PD was significantly related to preoperative characteristics (including age ≥ 70 years, white blood cell count >10 × 10^9^/L, and haemoglobin level < 90 g/L), intraoperative variables (such as operative time > 300 min), postoperative AKI status, and postoperative complications (including CR‐POPF, BL, IL, PPH grades B and C, and intra‐abdominal infections). The multivariate logistic regression analysis revealed that age ≥ 70 years (OR: 4.08; 95% CI: 1.56–10.64; *p* = 0.004) was the only independent preoperative risk factor and that postoperative complications such as CR‐POPF and BL were not significantly associated with 30‐day mortality. Postoperative complications such as IL (OR: 11.60; 95% CI: 2.66–50.71; *p* = 0.001), PPH grades B and C (OR: 11.25; 95% CI: 3.60–35.13; *p* < 0.001), and intra‐abdominal infections (OR: 3.46; 95% CI: 1.22–9.87; *p* = 0.020) still showed significant differences. Furthermore, postoperative AKI (OR: 2.87; 95% CI: 1.09–7.58; *P* = 0.034) was a new independent risk factor for 30‐day mortality.

### 3.6 Acute kidney injury's impact on long‐term survival outcomes

**FIGURE 1:**
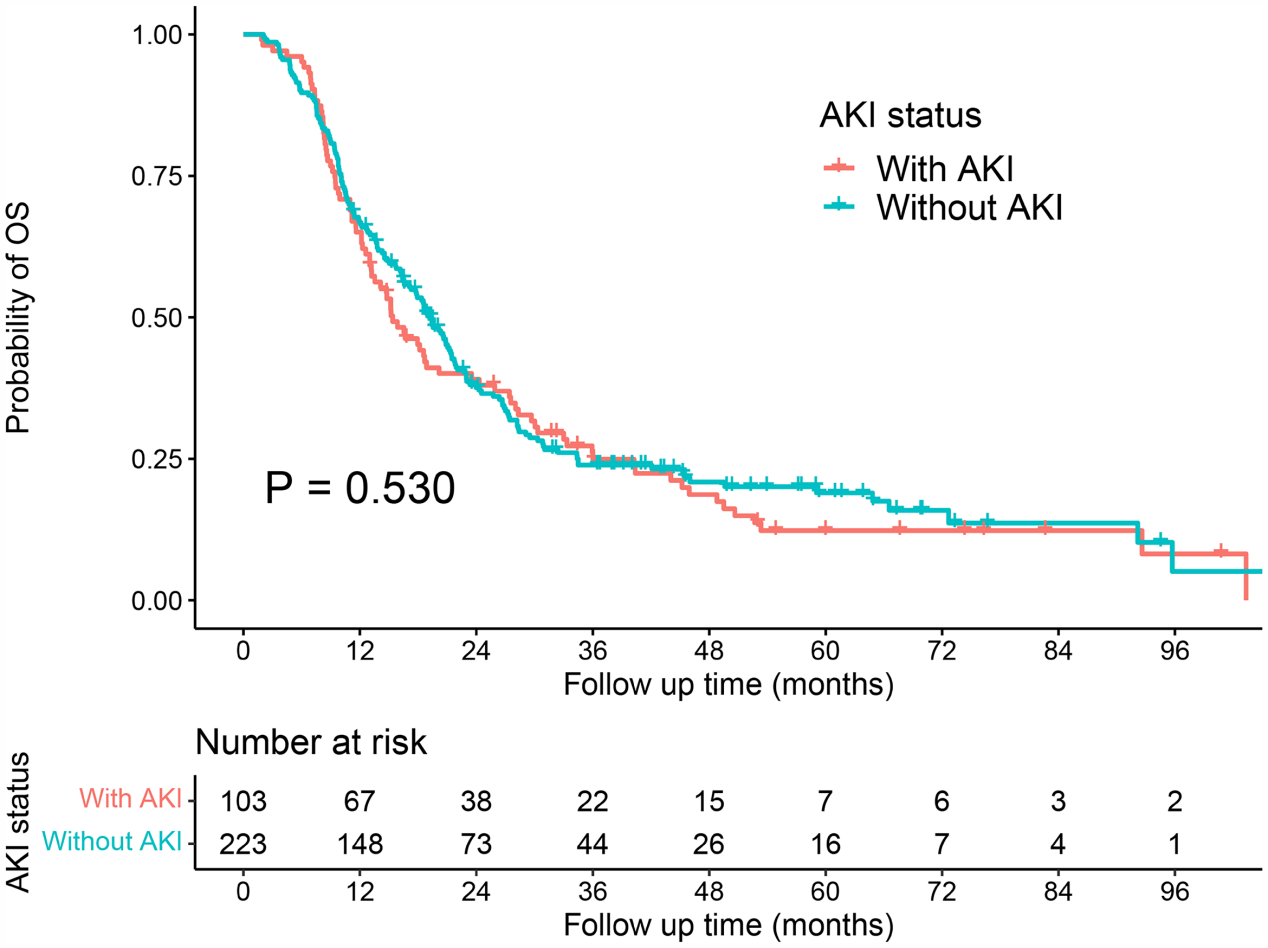
Description: Kaplan–Meier analysis of long‐term survival outcomes of patients with or without postoperative acute kidney injury (AKI) after pancreaticoduodenectomy for pancreatic ductal adenocarcinoma after propensity score matching. Methodology: The ‘survivfit’ function of the R software package ‘survival’ was used to analyse the difference in prognosis between the ‘With AKI’ group and the ‘Without AKI’ group. The significance between different groups was evaluated using the log‐rank test. Findings: After a median follow‐up time of 17 months, the long‐term overall survival outcomes of the ‘With AKI’ and ‘Without AKI’ groups were comparable (median of 14 months vs. 18 months, *p* = 0.53)

A total of 27 of 1312 patients died within 30 days after PD, and the remaining 1285 patients were followed up. Throughout the study until the last follow‐up, 946 (73.6%) patients died, and 6 were lost. During a median follow‐up of 17 months, the KM analysis revealed no difference (*p* = 0.53) in the long‐term OS of the AKI and non‐AKI groups after PSM (Figure 1); the median OS times were 14 and 18 months, respectively.

## 4 DISCUSSION

During this study, the potential risk factors for and impact of postoperative AKI after PD for PDAC were analysed using the KDIGO diagnostic criteria. We observed that TBil >250 μmol/L, eGFR <60 ml/min/1.73 m^2^, and intraoperative estimated blood loss >1000 ml were risk factors for AKI development. Furthermore, after PSM to reduce selection bias of baseline preoperative and intraoperative characteristics, we observed an increase in major complications and 30‐day mortality of the AKI group, and postoperative AKI did not affect the long‐term survival outcomes. These results suggest that postoperative AKI should be highlighted due to its strong impact on postoperative morbidity and mortality, and clinicians should attach great importance to developing AKI and perioperative management.

According to several reports on PD and other abdominal surgeries, eGFR^26^, ^27^ <60 ml/min/1.73 m^2^ indicates chronic kidney disease and is an independent predictor of AKI due to its direct effect on the kidney structure. The decrease in renal function and the inability to maintain the glomerular filtration rate is an essential aetiology of AKI. Secondly, changing arterial pressure and volume status during high‐risk surgeries like PD may initiate a series of systemic and local processes. A prolonged intraoperative period and postoperative hypotension could also explain the increased intraoperative blood loss as a prerenal factor for AKI occurrence. Additionally, reduced mean pressure due to great blood loss can lead to tissue hypoperfusion, organ hypoxia, and renin‐angiotensin‐aldosterone system disorder, which leads to the further development of AKI.^17^ Thirdly, we also identified TBil >250 μmol/L as another independent predictor that correlated with severe hyperbilirubinemia and obstructive jaundice.^28^ A high level of released serum proinflammatory cytokines and intestinal barrier function impairment caused by obstructive jaundice can lead to systemic or portal endotoxemia, worse nutritional status, and liver function disorder. Additional internal environment disorders such as digestive disorders, biliary infections, and immune reactions^28^ can lead to organ failure and explain the mechanism of sepsis‐associated and major surgery‐associated AKI development.^17^

Until now, many studies have assessed postoperative AKI and its association with morbidity and mortality after other surgeries. The overall AKI rate during this research was 10.7%, which corresponds with those reported for other major abdominal surgeries.^12^, ^26^, ^29^ Furthermore, our study revealed increased short‐term morbidity and mortality rates for patients with a higher AKI severity stage, consistent with the findings of several previous studies.^14^, ^30^ Various studies have revealed how AKI harms other organs and systems; nonetheless, few studies have discussed the potential mechanisms of digestive system impairment after PD. From our perspective, there are several major explanations. First, AKI creates disordered nutrition. Further accelerated proteolysis caused by AKI leads to the abnormal growth of anastomotic stomas, thus causing pancreatic fistulas, biliary and gastrointestinal leaks. Second, accumulated serum waste, such as the SCr, promotes the release of various inflammatory cytokines that can affect immune responses and cause infection or endotoxemia after PD. Finally, the inflammatory cytokines can also injure the vascular endothelium, and decreased renal function because of AKI causes liver function disorder, leading to impaired coagulation. In addition to hemodynamic changes, mucosal erosion and postoperative stressed state increase haemorrhage risks.

In our study, we also assessed the long‐term prognostic value of postoperative AKI after PD for PDAC. Several studies of long‐term survival outcomes after cardiac surgery^31^, ^32^, ^33^ indicated worse effects caused by haemodynamic changes in the cardiovascular system caused by AKI. In contrast, the mechanism seems indirect in cancer‐related abdominal surgery. Similar studies of hepatectomy for hepatocellular carcinoma included different results from various centres. The study by Lim^14^ of 457 consecutive patients showed no differences in long‐term survival outcomes; however, a recent study of 930 patients^34^ revealed a contrasting conclusion, with decreased 1‐, 3‐, and 5‐year OS rates in the AKI group. The results of our analysis are convincing due to the extremely large sample size and PSM. Our research presented similar OS rates with no significant difference between patients who did and did not develop AKI after PD for PDAC. Therefore, postoperative AKI did not predict long‐term survival outcomes.

The strong correlation between AKI development and adverse events postoperatively highlights the importance of early identification, prevention, and treatment. AKI is sometimes covert, and the impairment it causes often presents as a systematic disorder and other organs' dysfunction, such as electrolyte disturbance and congestive heart failure. Furthermore, decreased urine output was a notable symptom. Abnormal biomarkers commonly clinically used, such as accumulated SCr and blood urea nitrogen, appear long after onset, thus contributing to its rare early identification. It is also easy to ignore AKI, especially because further calculations are required to determine the change in the SCr level pre‐ and postoperatively according to the KDIGO criteria. Therefore, it is essential to find new diagnostic methods to prevent the mitigation of further injury. Biomarkers such as neutrophil gelatinase‐associated lipocalin^35^ and cystatin C^36^ have been investigated, and their clinical applications are being considered. To improve short‐term outcomes, there are other suggestions. First, according to our research, approximately 80% of AKI cases occurred within the first 2 days postoperatively. During this period, perioperative management advice regarding drug use and fluid therapy for potential high‐risk patients, especially those with a low eGFR, high TBil level, and high volume of intraoperative blood loss, should be obtained from nephrologists. Second, during the hospital stay, volume expansion, diuretics use, vasoactive drug administration, and nephrotoxic drug avoidance^25^, ^37^ should be carefully considered.

This study had several limitations. First, selection bias was unavoidable and logistic regression analysis depended greatly on function form settings, which could cause misspecifications. Therefore, we applied PSM as a two‐step verification. PSM had its advantages as it could show an obvious causal association. In our study, all confounding pre‐ and intraoperative factors were eliminated and AKI's effects on morbidity and mortality were directly evaluated. However, there were still several problems. PSM could not overcome all endogenous problems because unobservable or missing variables still existed. Furthermore, PSM was not a real ‘experiment’ and the efficacy would be greatly affected when common support was not achieved. Thus, the created cohorts could not completely represent the whole population. Second, important clinical data, such as urine output and blood pressure, along with their intraoperative and postoperative values, were incomplete or absent. Moreover, the timing and usage of intraoperative and postoperative volume resuscitation were also important, but achieving a consensus regarding perioperative management was challenging and varied from one institution to another. Third, renal function values were absent during our follow‐up. The renal function could eventually completely recover before discharge in over half of the patients during several previous studies,^14^, ^30^ the remaining patients who experienced partial or no recovery can develop chronic kidney disease without monitoring and treatment after a prolonged period. Furthermore, 6.3% of the patients with AKI in our study may have had pre‐existing kidney diseases, reducing the number of patients who had completely impaired renal function due to PD. These patients could not directly impact the long‐term survival outcomes. Finally, in this study, we focused on the impact of AKI on adverse events because most AKI cases occurred within the first 2 days postoperatively, and common complications, such as CR‐POPF and PPH grades B and C, often occurred after this period. Considering the sequence of occurrence, AKI may impact morbidities, and there are also interactions among the complications. Thus, the multivariate regression model revealing AKI's independent predictive effects on 30‐day mortality may not seem as accurate as expected. To our knowledge, it is challenging to solve this due to the design and restricted statistical methods. Moreover, eliminating collinearity between factors and identifying the independent predictive effect remain a global challenge. Therefore, to compensate for the shortage of logistic regression methods and provide more convincing results, PSM was applied in this study as a two‐step verification for the AKI and 30‐day mortality association. Furthermore, the large sample size also contributed to reducing the confounding interactions, and our centre had one of the world's highest volumes.

Finally, some unique insights about postoperative AKI were highlighted: 94.3% of patients with AKI in our cohort experienced stage 1 or 2 AKI, and the other 5.7% experienced stage 3. All the patients with AKI received slight clinical interventions such as fluid control or only observation. In our cohort, renal replacement therapy and ICU transfer were unnecessary as AKI did not affect the rehabilitation process within the first week postoperatively. Thus, AKI, defined by the KDIGO criteria, differed from the traditional ‘acute renal failure.’ It can be considered a new ‘conception’ instead of a ‘complication,’ and the significant SCr level change can be a biochemical indicator like postoperative hypoalbuminemia. Considering the predictive effect of AKI on postoperative morbidity and mortality, this new ‘conception’ and postoperative SCr level should stimulate doctors' attention.

## 5 CONCLUSIONS

Our study represents the first investigation of postoperative AKI after PD for PDAC. Based on the KDIGO criteria, AKI is frequent and significantly impacts 30‐day mortality. Postoperative AKI also increases the incidence of short‐term morbidity; however, it does not affect long‐term survival outcomes. Therefore, perioperative management should be performed with better consideration, especially for patients with high‐risk factors such as a high TBil level, decreased renal function, and a high volume of intraoperative blood loss.

## AUTHOR CONTRIBUTIONS

**Yuchen Ji:** Conceptualization (equal); data curation (equal); formal analysis (equal); methodology (equal); writing – original draft (equal). **Yiran Zhou:** Conceptualization (equal); formal analysis (equal); methodology (equal); writing – review and editing (equal). **Ziyun Shen:** Data curation (equal); formal analysis (equal); writing – review and editing (equal). **Haoda Chen:** Formal analysis (equal); methodology (equal); validation (equal). **Shulin Zhao:** Data curation (equal); resources (equal); software (equal); validation (equal). **Xiaxing Deng:** Conceptualization (equal); project administration (equal); resources (equal); supervision (equal). **Baiyong Shen:** Conceptualization (equal); methodology (equal); project administration (equal); supervision (equal); writing – review and editing (equal).

## CONFLICT OF INTEREST

The authors have no conflicts of interest to declare.

## FUNDING STATEMENT

None.

## ETHICAL APPROVAL STATEMENT

This study conformed to the provisions of the Declaration of Helsinki (as revised in 2013). All the patients included signed informed consent forms and agreed to data collection. It was approved by the institutional review board of Shanghai Ruijin Hospital.

## Supporting information


Table S1.

## Data Availability

The data supporting the findings of this study are available from the corresponding authors upon reasonable request.
