# Supplementary material for: Risk factors for and prognostic values of postoperative acute kidney injury after pancreaticoduodenectomy for pancreatic ductal adenocarcinoma: A retrospective, propensity score‐matched cohort study of 1312 patients
Source: Cancer Med. 2022 Dec 15;12(7):7823–34. doi: 10.1002/cam4.5543 (PMC10134349; doi:10.1002/cam4.5543)
Supplement: Supplementary file 1 — Table S1. [file CAM4-12-7823-s001.doc]

Table S1. Univariate and multivariate analyses of risk factors for major complications

| Risk factors | Univariate analysis |  |  | Multivariate analysis |  |
| --- | --- | --- | --- | --- | --- |
|  | OR (95% CI) | P-value |  | OR (95% CI) | P-value |
| Age≥70 years | 1.48 (0.92-2.38) | 0.104 |  |  |  |
| Male gender | 1.27 (0.79-2.06) | 0.323 |  |  |  |
| BMI＞28 kg/m^2 | 1.08 (0.38-3.04) | 0.891 |  |  |  |
| ASA class≥III | 0.62 (0.28-1.37) | 0.236 |  |  |  |
| Diabetes mellitus | 3.62 (2.30-5.71) | <0.001 |  | 3.57 (2.25-5.67) | <0.001 |
| Hypertension | 1.06 (0.67-1.68) | 0.791 |  |  |  |
| Cardiovascular disease | 0.83 (0.35-1.95) | 0.666 |  |  |  |
| Smoke | 1.67 (1.05-2.66) | 0.030 |  | 1.53 (0.86-2.71) | 0.147 |
| Alcohol | 1.72 (1.05-2.82) | 0.033 |  | 1.18 (0.63-2.19) | 0.604 |
| WBC＞10×10^9/L | 2.49 (1.19-5.23) | 0.016 |  | 2.09 (0.95-4.57) | 0.066 |
| Hb＜90 g/L | 1.75 (0.61-5.05) | 0.300 |  |  |  |
| TBil＞250 μmol/L | 1.88 (0.87-4.05) | 0.109 |  |  |  |
| ALB＜35 g/L | 1.21 (0.73-2.03) | 0.460 |  |  |  |
| Baseline sCr＞133 μmol/L | 1.68 (0.21-13.38) | 0.627 |  |  |  |
| eGFR＜60 ml/min/1.73 m^2 | 1.38 (0.41-4.59) | 0.602 |  |  |  |
| Preoperative biliary drainage | 1.37 (0.86-2.17) | 0.187 |  |  |  |
| Surgical type, RPD | 1.05 (0.56-1.97) | 0.886 |  |  |  |
| Venous resection | 1.60 (0.95-2.71) | 0.078 |  |  |  |
| Operative time＞300 min | 1.31 (0.83-2.05) | 0.243 |  |  |  |
| Estimated blood loss＞1000 ml | 1.90 (0.79-4.58) | 0.151 |  |  |  |
| Intraoperative transfusion＞1000 ml | 1.54 (0.96-2.46) | 0.073 |  |  |  |
| Postoperative AKI | 3.18 (1.87-5.41) | <0.001 |  | 3.06 (1.76-5.32) | <0.001 |

BMI, body mass index; ASA, American Society of Anesthesiologists; WBC, white blood cell; Hb, hemoglobin; TBil, total bilirubin; ALB, albumin; sCr, serum creatinine; eGFR, estimated glomerular filtration rate; RPD, robotic pancreaticoduodenectomy; AKI, acute kidney injury
